# Cellulase and Xylanase Production by *Penicillium echinulatum* in Submerged Media Containing Cellulose Amended with Sorbitol

**DOI:** 10.1155/2013/240219

**Published:** 2013-08-22

**Authors:** Carla Eliana Todero Ritter, Marli Camassola, Denise Zampieri, Mauricio Moura Silveira, Aldo José Pinheiro Dillon

**Affiliations:** Enzymes and Biomass Laboratory, Institute of Biotechnology, University of Caxias do Sul, Rua Francisco Getúlio Vargas 1130, 95070-560 Caxias do Sul, RS, Brazil

## Abstract

The present work investigated the use of sorbitol as a soluble carbon source, in association with cellulose, to produce cellulases and xylanases in submerged cultures of *Penicillium echinulatum* 9A02S1. Because cellulose is an insoluble carbon source, in cellulase production, there are some problems with rheology and oxygen transfer. The submerged fermentations containing media composed of 0, 0.25, 0.5, 0.75, and 1% (w/v) sorbitol and cellulose that were added at different times during the cultivation; 0.2% (w/v) soy bran; 0.1% (w/v) wheat bran; and a solution of salts. The highest filter paper activity (FPA) (1.95 ± 0.04 IU*·*mL^−1^) was obtained on the seventh day in the medium containing 0.5% (w/v) sorbitol and 0.5% (w/v) cellulose added 24 h after the start of cultivation. However, the CMCases showed an activity peak on the sixth day (9.99 ± 0.75 IU*·*mL^−1^) in the medium containing 0.75% (w/v) sorbitol and 0.75% (w/v) cellulose added after 12 h of cultivation. The xylanases showed the highest activity in the medium with 0.75% (w/v) sorbitol and 0.25% (w/v) cellulose added 36 h after the start of cultivation. This strategy enables the reduction of the cellulose concentration, which in high concentrations can cause rheological and oxygen transfer problems.

## 1. Introduction

Lignocellulosic biomass has been projected to be one of the main resources for economically attractive bioethanol production, and enzymatic hydrolysis is the most potent alternative process for the saccharification of its polymers. Cellulase is an enzyme complex capable of hydrolyzing cellulose into glucose molecules [1], and xylanases degrade xylan, the main carbohydrate present in some hemicelluloses, into xylose [2]. Although cellulases and xylanases have several industrial uses, the greatest potential use of these enzymes is in the enzymatic hydrolysis of lignocellulosic materials to produce second-generation ethanol [3]. 

The cellulase complex has three major hydrolases: the endo-*β*-1,4-glucanases (EG I, EG II, EG III, EG IV, and EG V; EC 3.2.1.4), which hydrolyze the glucosidic bonds randomly in cellulose fiber; the exo-*β*-1,4-glucanases or cellobiohydrolases (CBH I and CBH II; EC 3.2.1.91), which act on the reducing and nonreducing ends of polymers, releasing cellobiose; and the *β*-1,4-glucosidases (BG I and BG II; EC 3.2.1.21), which hydrolyze oligosaccharides and cellobiose into glucose [4]. The xylanolytic complex capable of hydrolyzing xylan is usually composed of several enzymes, such as *β*-1,4-endoxylanase, *β*-xylosidase, *α*-L-arabinofuranosidase, *α*-glucuronidase, acetyl xylan esterase, and the phenolic, ferulic, and *ρ*-coumaric esterases, that act cooperatively to convert hemicellulose to monosaccharides [5].

The production of the cellulolytic complex is induced by the presence of cellulose or several oligosaccharides and disaccharides, such as sophorose, *δ*-cellobiono-1,5-lactone, and gentiobiose [6–10], which are some of the saccharides resulting from cellulose itself. In addition to inducing cellulase production, cellulose induces the production of xylanases, which is attributed to the fact that the regulator of cellulase production, ACEII, also affects the regulation of xylanase production [11].

Xylobiose and xylan were shown to induce specific xylanases of *Trichoderma reesei*, whereas sophorose induced the expression of enzymes active on both xylan and cellulose [12]. The optimization of the cultivation media favors microorganism growth and increases the enzyme concentration in less time, thereby reducing the costs [13, 14]. During the cellulase production processes, the secretion of these enzymes is greatly impaired due to rheological and oxygen transfer problems caused by the high concentration of the cellulose used as an inducing substrate, mainly because cellulose is insoluble [15]. 

The microorganisms with the potential to produce cellulases include *Penicillium echinulatum* mutants with high enzyme production [16–18], and its enzyme complex shows good stability at 50°C, which is an important condition for using these enzymes in the enzymatic hydrolysis of cellulose [19]. This enzymatic complex also presents a higher *β*-glucosidase activities in relation to FPA compared with the enzyme complex of *T. reesei* [20]. Additionally, studies with the *P. echinulatum* 9A02S1 strain have been conducted with the purpose of associating lactose with cellulose to produce cellulases, but when this disaccharide was used as the only carbon source, no cellulase secretion occurred [18], which is in contrast to the results found in *T. reesei *[21].

However, studies with *T. reesei* have shown that the polyols glycerol and sorbitol allow growth without causing catabolic repression. In practice, sorbitol can be considered a neutral carbon source for cellulase expression [21]. A neutral carbon source does not contribute to the expression of repressor or activator proteins. However, there are no studies using these substrates for the production of cellulases and xylanases by *P. echinulatum*. 

Due to the importance of totally or partially replacing cellulose with soluble carbon sources in the cultivation medium to favor the rheology and promote cellulase production, the aim of the present work was to evaluate the use of sorbitol as a soluble carbon source in association with cellulose in submerged cultivation for the production of cellulases using the strain *P. echinulatum* 9A02S1. The xylanase activity was also determined because for the application of these enzymes to the hydrolysis of biomass, the presence of xylanases contributes to an increase in the yield of sugar liberation. The suitable time to add cellulose to the media to achieve higher enzyme production was also investigated. Recently, the interest in new ethanol-producing microorganisms has increased, and the bacterium *Zymomonas mobilis* represents a good alternative to currently used microorganisms. Sorbitol can be economically produced because *Zymomonas mobilis* can be used to produce both sorbitol and gluconic acid using sucrose or mixtures of glucose and fructose [10]. 

## 2. Materials and Methods

### 2.1. Microorganism

The mutant *P. echinulatum* strain 9A02S1 (DSM 18942) was used throughout this study. The strain was obtained by exposing the wild-type *P. echinulatum* strain 2HH to different mutagenic agents [17]. These strains are stored in the culture collection of the Laboratory of Enzyme and Biomass, University of Caxias do Sul, Caxias do Sul, RS, Brazil. 

### 2.2. Cultivation

The submerged fermentations were performed in 500 mL Erlenmeyer flasks containing 100 mL of medium composed of 0, 0.25, 0.5, 0.75, and 1% (w/v) sorbitol; 0.2% (w/v) soy bran; 0.1% (w/v) wheat bran; 0.14% (w/v) KNO_3_; and a 5% (v/v) 20X concentrated mineral salt solution containing the following salts (g L^−1^): KH_2_PO_4_, 20; CO(NH_2_)_2_, 3; MgSO_4_·7H_2_O, 3; CaCl_2_, 3; FeSO_4_·7H_2_O, 0.050; MnSO_4_·H_2_O, 0.0156; ZnSO_4_·7H_2_O, 0.014; and CoCl_2_, 0.020. The quantities of cellulose and sorbitol were chosen according to previous results. Some experiments were performed without soy bran or wheat bran supplementation, as previous work with this strain showed that soy bran could replace a protein source and show a higher FPA when wheat bran was added to the media. 

Crystalline cellulose (Celuflok (Cotia, SP, Brazil)) was added to the medium at 0, 12, 24, 36, or 48 h of cultivation time. The flasks were inoculated with a 1 × 10^5^ conidia mL^−1^ suspension in a 0.9% NaCl solution and maintained under reciprocal agitation at 180 rpm and 28°C. All cultures were grown in triplicate.

### 2.3. Enzyme Activity

The enzyme activity was assayed on filter paper (FPA), and CMCase was assayed according to the method of Ghose [22] using carboxymethylcellulose. The *β*-glucosidase activity was measured according to the method of Chahal [23] using salicin. The reducing sugar was estimated according to the method of Miller [24]. The xylanase activity was measured by the method of Bailey et al. [25] using oat spelt xylan. One international unit (IU) of enzyme activity was defined as the amount of enzyme required to release 1 *μ*moL of reducing sugar from the appropriate substrate per minute under the assay conditions. 

The enzymatic activities were not determined for the first and second days of cultivation because the inoculums were performed with spores. Under these conditions, low enzyme titers were obtained during the first two days of cultivation.

### 2.4. Zymograms

Modified SDS-polyacrylamide gel electrophoresis (SDS-PAGE) was performed, as described by Laemmli [26]. The samples were prepared with the same enzymatic broth volume for each substrate. The bands on the gel were used for visualization of the CMCase and xylanase activities [27]. The proteins were separated on a 12% separating gel containing 0.15% carboxymethylcellulose or 0.1% oat spelt xylan. The gel was washed at room temperature, and the wash solution, which contained 50 mM sodium citrate buffer (pH 4.8) and 25% isopropanol, was changed twice to remove the SDS. Then, the gel was transferred to a 50 mM sodium citrate buffer (pH 4.8) for 30 min and incubated at 50°C for 10 min. The gels were stained in 0.2% Congo red for 30 min and decolorized in a 1 M NaCl solution. Clear bands against the red background indicated the breakdown of carbohydrates.

### 2.5. Statistical Treatment

The results were submitted to an analysis of variance with Tukey's post hoc test for a *P* < 0.05 using the Prism GraphPad program (Graph Pad, San Diego, CA, USA).

## 3. Results and Discussion

Although cellulose is a carbon source that induces the production of cellulases and xylanases in *T. reesei* [21] at high concentrations, a condition necessary to achieve high enzyme levels [15], problems can arise in the transfer of oxygen through the cultivation medium, causing negative repercussions on growth and enzyme production [15, 28]. In addition, the presence of cellulose in the medium can reduce the quantity of free cellulases because these enzymes tend to become adsorbed to their substrates [29].

In the present work, the polyol sorbitol, a soluble carbon source that can be converted into fructose by L-iditol 2-dehydrogenase or by sorbitol dehydrogenase and can be used in microbial growth [30], was assayed for its ability to improve the production of cellulases and xylanases in association with cellulose during the submerged cultivation of *P. echinulatum*. 

The production of cellulases and xylanases by *P. echinulatum* 9A02S1 was assessed in media formulated with different sorbitol concentrations, which were present from the beginning of cultivation, combined with different cellulose concentrations added to the media at various times. 

**Figure 1:**
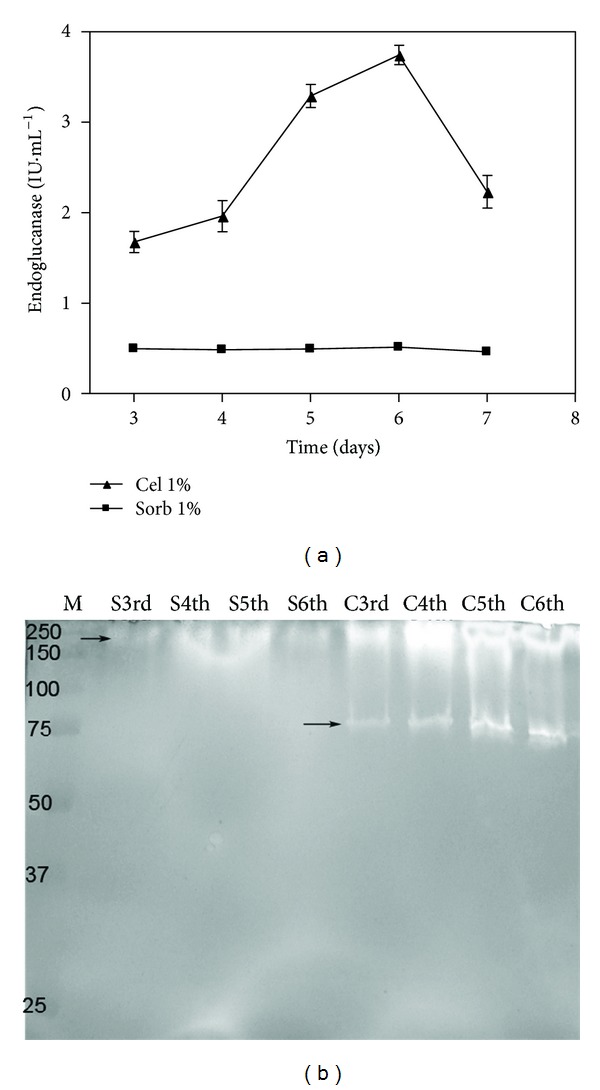
CMCase activities (a) and zymogram (b) of submerged cultures of *Penicillium echinulatum* 9A02S1 in media containing 1% (w/v) cellulose or sorbitol. The proteins were separated on a 12% polyacrylamide gel. M: standard protein molecular weights in kDa. The capital letters S and C indicate the substrates sorbitol and cellulose, respectively. The ordinal numbers indicate the times at which the collections were performed.

The condition in which only sorbitol was used as a carbon source (Figure 1(a)) showed low enzymatic titers (approximately 0.5 IU·mL^−1^) of CMCase. In contrast, in the medium with cellulose, a peak of CMCase activity was obtained on the sixth day of cultivation, reaching values of 3.74 IU·mL^−1^. This value was low compared with the other data obtained for *P. echinulatum* or other fungi, as the medium that was used was very poor in nutrient. In the zymograms, the CMCase activity detection (Figure 1(b)) in the cultures formulated with only sorbitol produced only one slight band with an apparent molecular mass of approximately 250 kDa. This band was also observed, even for a sample with 100 times the concentration of a sample obtained from the cultivations with cellulose in the medium, in which two bands of apparent molecular masses of approximately 250 kDa and 80 kDa were observed (Figure 1(b)).

This observation may suggest that the smaller CMCase originated from cleavage of the larger form. Future experiments involving the sequencing of these proteins may clarify these findings. However, the absence of CMCase activity (Figure 1(a)) and CMCase bands in the zymogram (Figure 1(b)) from the cultures with sorbitol alone clearly showed that this polyol was not a CMCase inducer in *P. echinulatum*, as has been shown previously for *T. reesei* [21].

**Figure 2:**
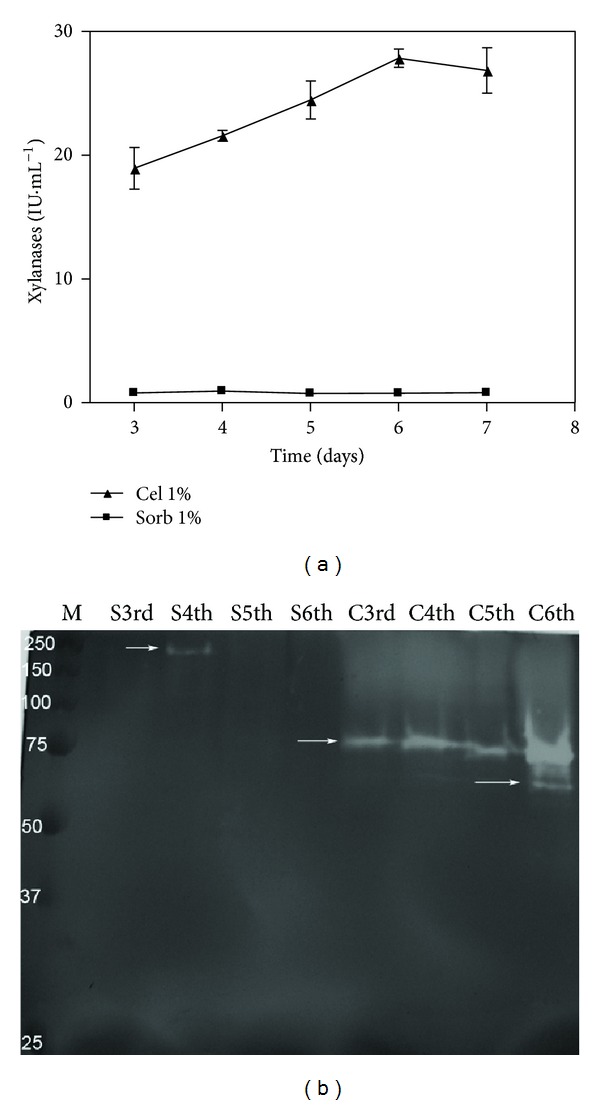
Xylanase activities (a) and zymogram (b) of the submerged cultures of *Penicillium echinulatum* 9A02S1 in media containing 1% (w/v) cellulose or sorbitol. The proteins were separated on a 12% polyacrylamide gel. M: standard protein molecular weights in kilodaltons. The capital letters S and C indicate the substrates sorbitol and cellulose, respectively. The ordinal numbers indicate the times at which the collections were performed.

Similar to the CMCase activity, low xylanase activity was observed from the cultivation with sorbitol as the only carbon source compared with the cultivation supplemented with cellulose (Figure 2(a)). However, in the gel activity (Figure 2(b)), the presence of a band with xylanolytic activity and an apparent molecular mass of approximately 240 kDa was observed on the third day of cultivation. Interestingly, the samples from the medium formulated with cellulose showed at least two bands with apparent masses of approximately 80 kDa and 60 kDa. The xylanase (240 kDa) found in the medium with sorbitol may be a constitutive enzyme, whereas the other xylanases found when cellulose was used as a carbon source may represent inducible adaptations. 

The observed differences in the xylanase activity (Figure 2(a)) and zymogram activity of the xylanases (Figure 2(b)) between the cultures supplemented with sorbitol and cellulose confirmed that cellulose can also induce xylanases in *P. echinulatum*, as has already been observed for *T. reesei* [11]. Therefore, the small number of xylanases found in the samples of the culture supplemented with sorbitol may suggest that sorbitol has a low potential for inducing xylanase or that these enzymes are constitutive and present at low concentrations, as has been observed for cellulases in *T. reesei *[31]. Interestingly, the xylanase secreted into the medium supplemented with sorbitol showed a molecular mass of approximately 240 kDa, whereas the forms of the xylanases obtained in the medium supplemented with cellulose showed molecular masses of approximately 80 kDa and 60 kDa. The xylanase (240 kDa) found when sorbitol was the only carbon source could be a constitutive or basal enzyme, whereas the other two (80 kDa and 60 kDa) may be induced by cellulose. 

This result suggested that different genes or posttranslational systems are involved in the expression of xylanases in *P. echinulatum*, depending on the carbon source that is available. Additionally, the 240 kDa protein may be an enzyme that can hydrolyze both carboxymethylcellulose and xylan.

In the culture composed of 1% (w/v) sorbitol (w/v) with wheat bran (0.1%) and soy bran (0.2%) as the protein sources (Table 1), low but measurable cellulase and xylanase activities were observed, and these activities were higher than those shown in Figure 1(a) (CMCase data) and Figure 1(b) (xylanase data). The increase in *β*-glucosidase activity from the 3rd to the 6th days may be due to cell lysis (Table 1). 

The main importance of wheat bran and soy bran as additives for cellulase production is that both are residues and are less expensive than other sources and that they can be used in medium formulation for cellulase secretion by *P. echinulatum.*

**Figure 3:**
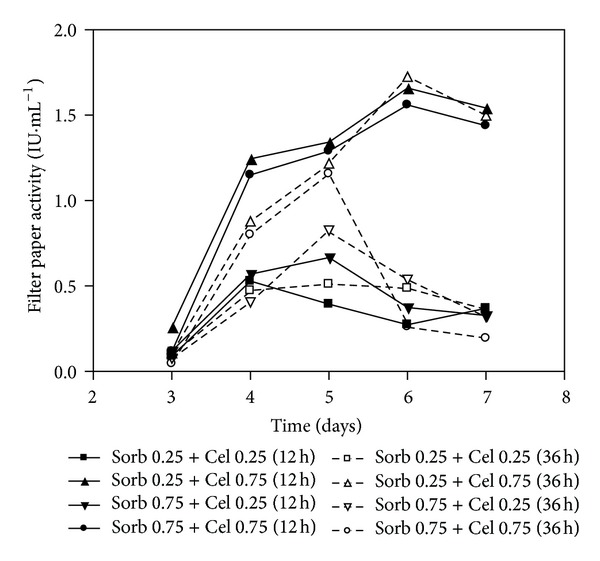
Filter paper activities of the submerged cultures of *Penicillium echinulatum* 9A02S1 in media containing sorbitol supplemented with cellulose at 12 and 36 hours of cultivation. The legend shows the concentration values of sorbitol and cellulose (% w/v), and the brackets indicate the times at which the cellulose was added to the cultivation.

**Figure 4:**
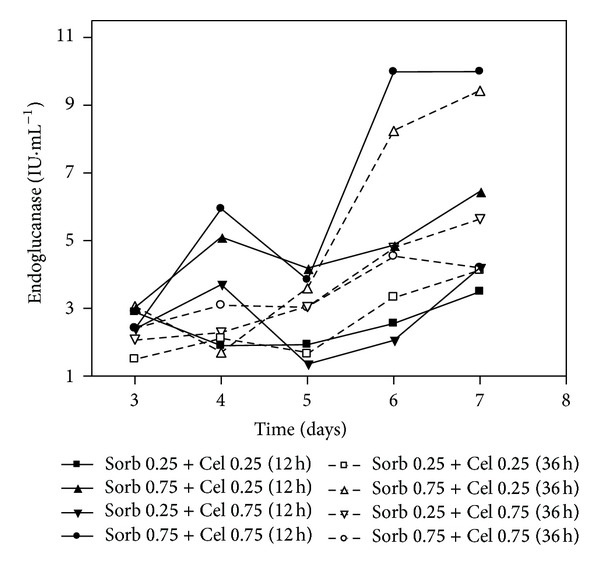
CMCase activity of the submerged cultures of *Penicillium echinulatum* 9A02S1 in media containing different concentrations of sorbitol and supplemented with cellulose after 12 and 36 hours of cultivation. The legend shows the concentration values of sorbitol and cellulose (% w/v), and the brackets indicate the times at which the cellulose was added to the cultivation.

**Figure 5:**
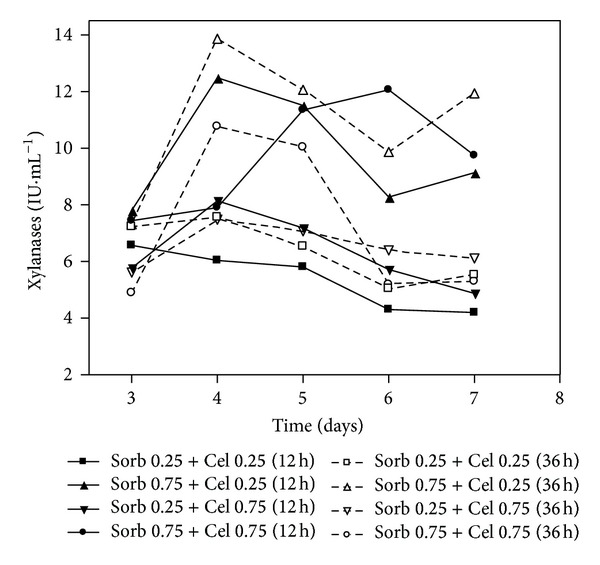
Xylanase activity of the submerged cultures of *Penicillium echinulatum* 9A02S1 in media containing different concentrations of sorbitol and supplemented with cellulose after 12 and 36 hours of cultivation. The legend shows the concentration values of sorbitol and cellulose (% w/v), and the brackets indicate the times at which the cellulose was added to the cultivation.

The data shown in Figure 3 (FPA), Figure 4 (CMCase), and Figure 5 (Xylanase) were obtained from cultivations in which the medium initially contained sorbitol at concentrations of 0.25% or 0.75% (w/v) that were later supplemented with cellulose at concentrations of 0.25% or 0.75% (w/v) after 12 or 36 hours of cultivation. These experiments were performed with supplementation with both soy bran (0.2%) and wheat bran (0.1%). In contrast to expectations, the culture Sorb 0.75 + Cel 0.75 (36 h) produced lower CMCase activity compared with the culture Sorb 0.75 + Cel 0.25 (36 h). This result may be due to the fungus's preferential consumption of the amorphous cellulose instead of the crystalline cellulose after the consumption of sorbitol, as the cellulose concentration was higher in the Sorb 0.75 + Cel 0.75 (36 h) medium than in the 0.75 + Cel 0.25 (36 h) medium, producing low titers of CMCases. The amorphous portions are components of cellulose that can be easily hydrolyzed. 

The initial concentrations of 0.25% sorbitol supplemented with 0.75% cellulose (Sorb 0.75 + Cel 0.75) resulted in higher FPA values, regardless of whether cellulose was added at 12 h (1.67 IU·mL^−1^) or 36 h (1.73 IU·mL^−1^) of cultivation. In contrast, cultures containing 0.25% sorbitol and supplemented with 0.25% cellulose showed lower FPA activities on the sixth and seventh days. 

The culture that contained an initial sorbitol concentration of 0.75% and that was supplemented with 0.75% cellulose after 36 h of cultivation (Sorb 0.75 + Cel 0.75 (36 h)) showed peak activity on the fifth day (1.16 IU·mL^−1^) but a marked decrease in activity afterwards. Although this decrease in enzymatic activity is commonly observed during cellulase production, this phenomenon is not completely understood. Some authors have suggested that the decrease in activity may be due to the effects of a protease on the stability of the cellulases [32], but the presence of inhibitors may also interfere. However, the culture initiated with 0.75% sorbitol and supplemented with cellulose (0.75%) at 12 h after the start of cultivation (Sorb 0.75 + Cel 0.75 (12 h)) reached FPA values of 1.57 ± 0.27 IU.mL^−1^. The data collected for the *β*-glucosidase activities were considered low (less than 0.2 IU·mL^−1^) and, therefore, are not shown. These results indicated that sorbitol can inhibit the production of *β*-glucosidase.

With respect to the CMCase activities (Figure 4), the cultures Sorb 0.75 + Cel 0.75 (12 h) and Sorb 0.75 + Cel 0.25 (36 h) showed the highest activities, as observed on the sixth and seventh days. The culture Sorb 0.25 + Cel 0.25 (12 h), although supplemented with cellulose at 12 h, showed low activity, possibly due to the lower concentration of cellulose. In agreement with the FPA data, the Sorb 0.75 + Cel 0.75 (36 h) cultivation also showed low enzymatic activity.

The highest xylanase activities were reported on the fourth day in the cultures Sorb 0.75 + Cel 0.25 (12 h) and Sorb 0.75 + Cel 0.25 (36 h) compared with the Sorb 0.25 + Cel 0.75 (12 h), Sorb 0.25 + Cel 0.75 (36 h), Sorb 0.75 + Cel 0.75 (12 h), and Sorb 0.75 + Cel 0.75 (36 h) cultures. This result suggested that higher xylanase activities can be obtained with only 0.25% cellulose added to 0.75% sorbitol (Figure 5).

The data obtained from the present work suggested that sorbitol is neither a strong inducer nor a strong repressor of cellulase production in *P. echinulatum* 9A02S1, as occurs in *T. reesei* [33]. The lack of repression by sorbitol was evident because the secretion of cellulases occurred in all media containing sorbitol with cellulose. A similar observation was made previously for *T. reesei*, in which sorbitol can be considered a carbon source that contributes to mycelial growth but does not act as repressor [28, 33, 34].

**Figure 6:**
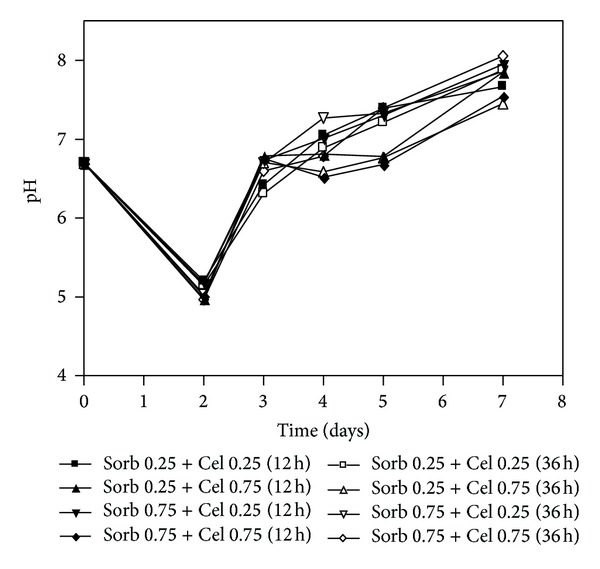
Variation of the pH values in the submerged cultures of *Penicillium echinulatum* 9A02S1 in media containing different concentrations of sorbitol and supplemented with cellulose after 12 h and 36 h of cultivation. The legend shows the concentration values of sorbitol and cellulose (% w/v), and the brackets indicate the times at which the cellulose was added to the cultivation.

The pH profiles showed a drop in the values at the beginning of the process for all cultures, but from the third day onward, there were similar increases in this parameter for all cultivations (Figure 6). The pH variation suggested growth uniformity in all cultures because the acid phase during cellulase production occurs due to the conversion of NH_3_ from (NH_4_)^+^, whereas the alkaline phase depends on the release of NH_3_ by the cell after the end of the growth stage [28].

**Figure 7:**
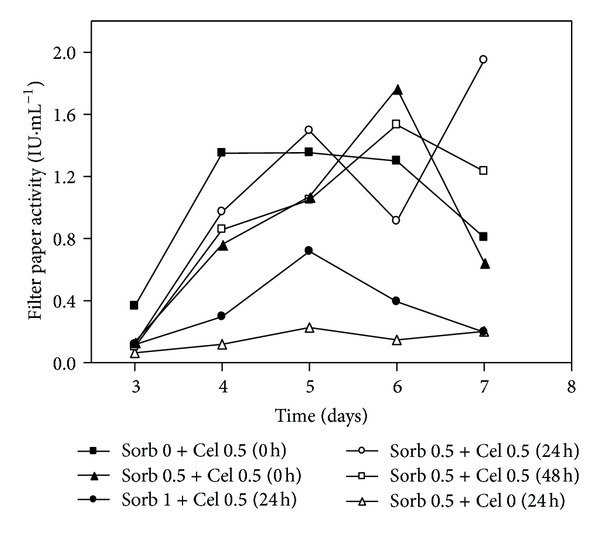
Filter paper activities of the submerged cultures of *Penicillium echinulatum* 9A02S1 in media with or without sorbitol and supplemented with cellulose at 0, 24, or 48 hours of cultivation. The legend shows the concentration values of sorbitol and cellulose (% w/v), and the brackets indicate the times at which the cellulose was added to the cultivation.

The results (Figures 3, 4, 5, 6, and 7) suggested that 0.75% sorbitol was unfavorable when cellulose 0.75% was used with respect to the secretion of the enzyme studied. Thus, 0.5% sorbitol may create good conditions, better than those of 0.75% sorbitol, when used in combination with cellulose. Another uncertainty about the time of the cellulose addition is whether it should be added at the start of cultivation. To address this question, in the next step, sorbitol (0.5 and 1%) supplemented with 0.5% cellulose at 0, 24, or 48 hours of cultivation was evaluated (Figure 7).

Figure 7 shows the cellulase production data, estimated from measuring the FPA, in the cultures in which sorbitol (0.5 or 1% (w/v)) was supplemented with 0.5% cellulose at 0, 24, and 48 hours of cultivation. Again, when sorbitol was the main carbon source (Sorb 0.5 + Cel 0), the induction of cellulase did not occur, or there was a very low FPA. Alternatively, when only cellulose (Sorb 0 + Cel 0.5 (0 h)) was used as the main carbon source, cellulase production occurred earlier (third day), as measured by FPA, and reached 1.35 ± 0.15 IU·mL^−1^ on the fourth day. The activity remained unchanged on the fifth day but decreased from the sixth day onwards, possibly due to exhaustion of the carbon source. These data suggested that cultivation with cellulose but without sorbitol induced a faster secretion of the cellulases. Although the mass of the fungus was not determined, mainly because cellulose is an insoluble carbon source, which makes mass determination difficult, the analysis of the enzyme activity in Figure 7 suggests that sorbitol encourages fungal growth during the beginning of the culture and that due to the subsequent presence of cellulose in the medium, a higher enzyme level was achieved because of the higher fungal mass. However, the cultivation medium with 1% sorbitol showed a negative effect on the production of enzymes. The same effect was observed when the media containing higher sorbitol concentrations (0.75%) were supplemented with 0.25% cellulose, which showed lower activities compared with cultures that began with only 0.25% sorbitol. A sorbitol concentration greater than 0.5% may be unfavorable for cellulase secretion in *P. echinulatum*. Sorbitol is not a repressive carbon source in *T. reesei* [21], but a study with the ascomycete *Melanocarpus *sp. showed the strong repression of *β*-glucosidase production when sorbitol was added to the CMC medium [11]. 

However, the medium containing 0.5% (w/v) sorbitol with 0.5% (w/v) cellulose added at the beginning of cultivation (Sorb 0.5+Cel 0.5 (0 h)) showed an FPA of only 0.76 ± 0.02 IU·mL^−1^ on the fourth day. High activity on the fifth day was also observed in the culture with 0.5% (w/v) sorbitol and 0.5% (w/v) cellulose added after 24 hours (Sorb 0.5+Cel 0.5 (24 h)), followed by a decrease on the sixth day and an increase on the seventh day, when the highest FPA (1.95 ± 0.04 IU·mL^−1^) was recorded. 

Comparing the FPA results for the cultures with 0.5% sorbitol, we observed that the addition of cellulose (0.5%) at 0, 24, or 48 h led only to minor differences in the enzyme activity on the fourth day. The addition of cellulose at the start of cultivation led to a greater productivity per day than when cellulose was added after 24 hours. Although the experiment was inconclusive because it was interrupted on the seventh day, the supplementation with cellulose after 48 hours (Sorb 0.5 + Cel 0.5 (48 h)) would likely show an activity peak only after the seventh day, with a greater effect on the productivity. Lower activities were observed in the cultures with media containing 1% sorbitol (Sorb 1.0 + Cel 0.5 (24 h)). It is important to mention that the FPA values in the present work are in line with those obtained using the *T. reesei* RUT C30 strain when 1% [34] or 0.75% carbon sources were used [42]. Other works have also used *T. reesei* RUT C30 and other substrates to produce FPA values (Table 2). 

Additionally, there could be a link between D-sorbitol utilization and cellulase production, at least for *T. reesei*, because D-sorbitol can be converted into L-sorbose by an NADP-dependent ketose reductase [43], and L-sorbose, in turn, can induce the production of cellulases in *T. reesei *[44].

**Figure 8:**
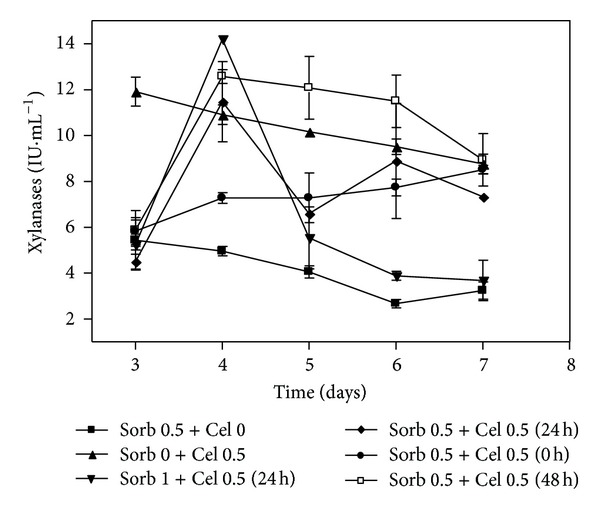
Variation of the xylanases in submerged cultures of *Penicillium echinulatum* 9A02S1 in media with or without sorbitol and supplemented with cellulose at 0, 24, or 48 hours of cultivation. The legend shows the concentration values of sorbitol and cellulose (% w/v), and the brackets indicate the times at which the cellulose was added to the cultivation.

Figure 8 shows the activities of the xylanases. The cultivation containing Sorb 0.5 + Cel 0 showed low xylanase activity. On the third day, all cultures containing sorbitol showed lower xylanase activities than the treatments containing only cellulose (Sorb 0 +Cel 0.5+ (24 h)), indicating that cellulose induced the production of this enzyme and that sorbitol did not stimulate faster production of xylanases.

In addition, the activity profile shown by Sorb 0.5 + Cel 0.5 (48 h) reached high enzyme levels and remained steady between the fourth and sixth days. 

The most important result presented in this work was the finding that 0.25% sorbitol supplemented with 0.75% cellulose (Sorb 0.75 + Cel 0.75) resulted in FPA values of 1.67 IU·mL^−1^or 1.73 IU·mL^−1^, depending on whether cellulose was added at 12 h or after 36 h of cultivation, respectively. The values found were even higher (1.95 ± 0.04 IU·mL^−1^) in the culture with 0.5% (w/v) sorbitol with the addition of 0.5% (w/v) cellulose after 24 hours (Sorb 0.5 + Cel 0.5 (24 h)). These FPA values found for 9A02S1 were similar to those obtained with only 1% cellulose [17, 19] using this same strain. FPA values between 1.5 and 2 UI·mL^−1^ were also found for the *T. reesei *RUT C30 strain [41]. 

In other words, at least for *P*. *echinulatum* cultured with 1% cellulose as the carbon source, FPA values similar to those obtained using only half of the cellulose concentration can be obtained when sorbitol is also employed. Additionally, the experiments showed that 24 h was the best time for the addition of cellulose to the medium containing 0.5% (w/v) sorbitol.

## 4. Conclusion

The major goal of this work was to demonstrate the potential of using a soluble carbon source, such as sorbitol, together with cellulose, an insoluble carbon source, to induce cellulase production in *P. echinulatum. *These alterations in the media composition resulted in a reduction in the cellulose concentration in the media without altering the cellulase production. This effect may be even more significant if higher volumes are used. Filter paper activities near 1.95 IU·mL^−1^ were obtained in the medium containing 0.5% (w/v) sorbitol and 0.5% (w/v) cellulose. Additionally, the experiments showed that 24 h was the best time for the addition of cellulose to the medium containing 0.5% (w/v) sorbitol. 

## Figures and Tables

**Table 1:** Enzymatic activity in the submerged cultivation of *Penicillium echinulatum* 9A02S1 in media containing 1% (w/v) sorbitol.

Cultivation time (days)	CMCase (IU·mL^−1^)	*β*-Glucosidase (IU·mL^−1^)	FPA (IU·mL^−1^)	Xylanase (IU·mL^−1^)
2	1.04	0.04	0.21	1.89
3	1.17	0.05	0.20	5.45
4	2.06	0.14	0.39	6.12
5	0.95	0.27	0.23	3.12
6	0.92	0.47	0.18	1.45

The media used in this experiment were supplemented with soy bran and wheat bran.

**Table 2:** Comparison of FPA production by fungi using different substrates.

Microorganism	Substrate and concentration (%, w/v)	Enzyme activity (FPU·mL^−1^)	Reference
*P. echinulatum *	Cellulose (0.5%) Sorbitol (0.5%)	1.95	This work
*Acremonium cellulolyticus* C-1	Solka Floc (5%)	34.6	[35]
*P. occitanis *Pol6	Avicel PH 101 (16%)	23	[36]
*T. reesei* QM6a	Hydrolyzed cellulose (12%)	11.8	[37]
*T. reesei *QM 9414	Cellulose (4.2%)	3.2	[38]
*T. reesei* RUT-C30	Hardwood pulp (25%)	57	[39]
*T. reesei* RUT-C30	Solka Floc BW 200 (10%)	26.2	[15]
*T. reesei* RUT-C30	Solka Floc (8%)	31	[40]
*T. reesei* RUT-C30	Fermented corn residue (5.3%)	1.9	[41]
